# Supplementary material for: The causal relationship between the human gut microbiota and pyogenic arthritis: a Mendelian randomization study
Source: Front Cell Infect Microbiol. 2024 Nov 26;14:1452480. doi: 10.3389/fcimb.2024.1452480 (PMC11629706; doi:10.3389/fcimb.2024.1452480)
Supplement: Supplementary file 1 [file DataSheet1.zip › mendelian test/ebi-a-GCST90027668.csv_forest.pdf]

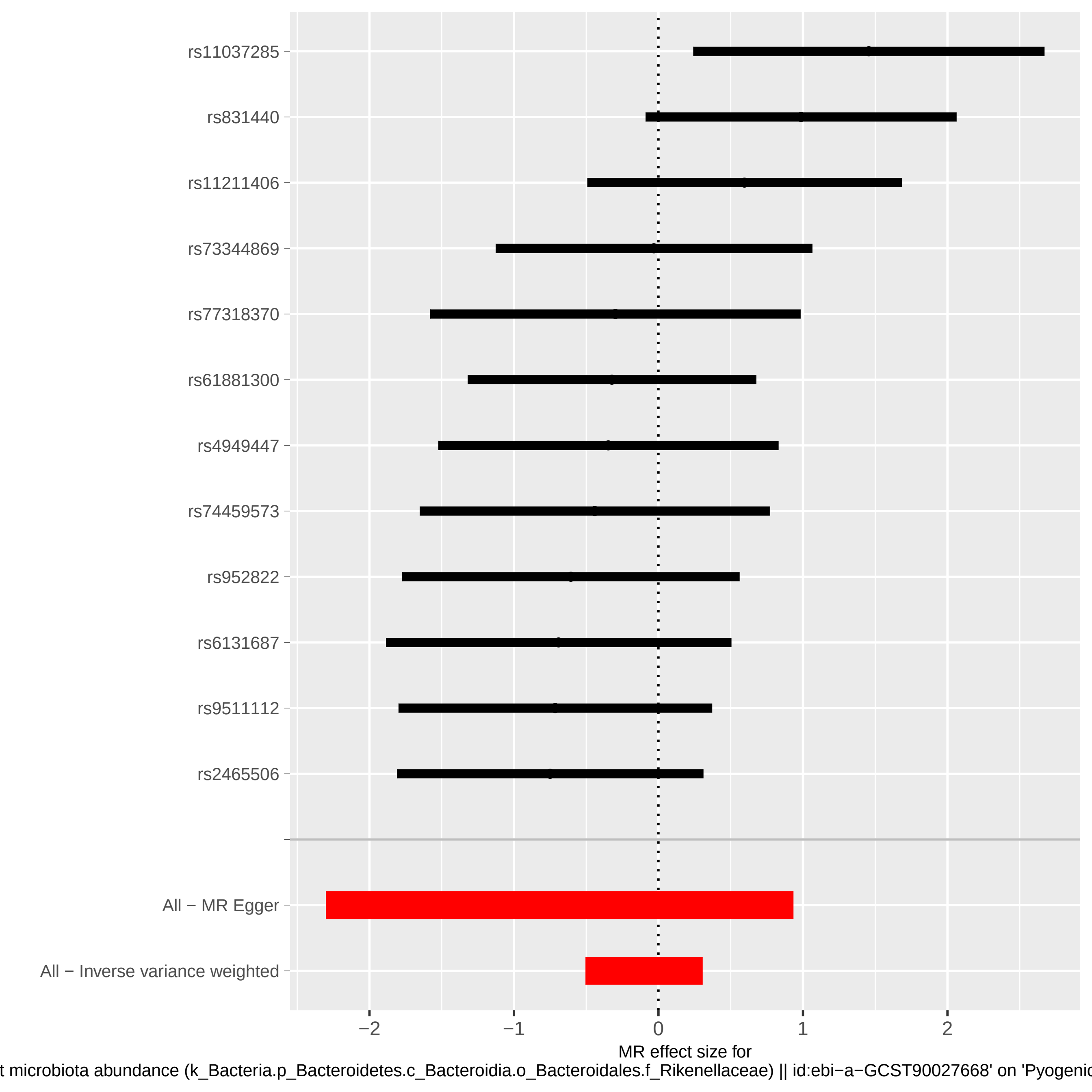
rs11037285
rs831440
rs11211406
rs73344869
rs77318370
rs61881300
rs4949447
rs74459573
rs952822
rs6131687
rs9511112
rs2465506
All − MR Egger
All − Inverse variance weighted
−2
−1
0
1
2
MR effect size for
t microbiota abundance (k_Bacteria.p_Bacteroidetes.c_Bacteroidia.o_Bacteroidales.f_Rikenellaceae) || id:ebi−a−GCST90027668' on 'Pyogenic
